# Dangerous Duplicity: The Dual Functions of Casein Kinase 1 in Parasite Biology and Host Subversion

**DOI:** 10.3389/fcimb.2021.655700

**Published:** 2021-03-22

**Authors:** Najma Rachidi, Uwe Knippschild, Gerald F. Späth

**Affiliations:** ^1^ Unité de Parasitologie moléculaire et Signalisation, Department of Parasites and Insect Vectors, Institut Pasteur and INSERM U1201, Paris, France; ^2^ Department of General and Visceral Surgery, Surgery Centre, Ulm University Hospital, Ulm, Germany

**Keywords:** casein kinase 1, apicomplexa, kinetoplastid, host-pathogen interactions, extracellular vesicles, drug target

## Abstract

Casein Kinase 1 (CK1) family members are serine/threonine protein kinases that are involved in many biological processes and highly conserved in eukaryotes from protozoan to humans. Even though pathogens exploit host CK1 signaling pathways to survive, the role of CK1 in infectious diseases and host/pathogen interaction is less well characterized compared to other diseases, such as cancer or neurodegenerative diseases. Here we present the current knowledge on CK1 in protozoan parasites highlighting their essential role for parasite survival and their importance for host-pathogen interactions. We also discuss how the dual requirement of CK1 family members for parasite biological processes and host subversion could be exploited to identify novel antimicrobial interventions.

## Introduction

Casein Kinase 1 (CK1) family members are serine/threonine signaling protein kinases, conserved in eukaryotes from protozoan to humans. They are involved in multiple cellular processes including protein trafficking, cell cycle regulation and apoptosis (Knippschild et al., 2005) and as a result display pleiotropic localisation and interact with various subcellular structures mediated by scaffolding proteins (Knippschild et al., 2005; Knippschild et al., 2014; Xu et al., 2019). To gain specificity, the activity of CK1 is regulated by different processes, including inhibitory autophosphorylation, phosphorylation by upstream kinases, protein-protein interactions, and subcellular sequestration (Knippschild et al., 2014). Defects in its regulation or mutations in its coding sequence are associated with important diseases such as cancer, and neurodegenerative diseases, including Alzheimer’s or Parkinson’s diseases (Knippschild et al., 2005; Schittek and Sinnberg, 2014; Jiang et al., 2018; Xu et al., 2019). Furthermore, there is substantial evidence suggesting that CK1 is associated with infectious diseases through the regulation of the host CK1 signaling pathways by intracellular pathogens in two different ways: either by directly subverting host CK1s (e.g. *Mycobacteria* and various viruses (Cegielska et al., 1994; Bhattacharya et al., 2009; Jayaswal et al., 2010; Sudha et al., 2012; Zhang et al., 2017)), or by exporting pathogen CK1 orthologs into the host cell (e.g. *Plasmodium* and *Leishmania* (Silverman et al., 2010; Dorin-Semblat et al., 2015)). Hence, CK1 is a key signaling molecule, instrumental for host-pathogen interactions. Here we review the current literature on CK1 in human parasitic pathogens and its dual functions, highlighting its essential role for parasite survival and the importance of released parasitic CK1s for host-pathogen interactions to propose CK1 as a major target for antimicrobial intervention.

## CK1 in Trypanosomatids

### CK1 Family Members in Leishmania

*Leishmania*, the causative agent of leishmaniasis, is transmitted to vertebrate hosts by the bite of a female sand fly, which injects promastigotes during its blood meal. These parasites are then phagocytised by macrophages, where they reside inside the phagolysosome and differentiate into proliferating amastigotes.

**Figure 1:**
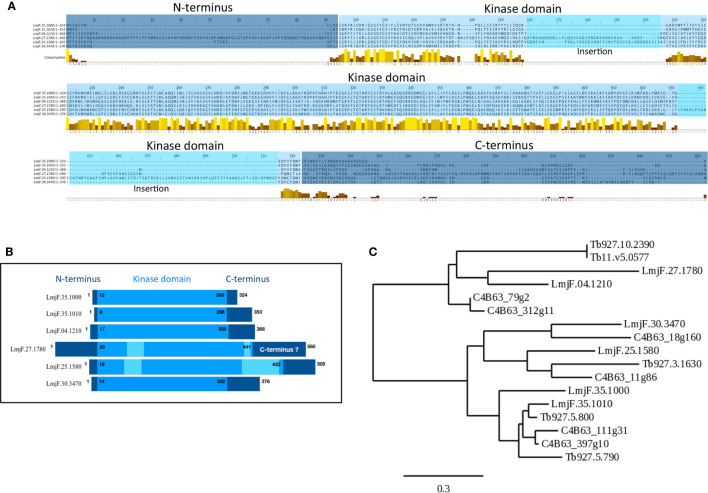
Visualization of conserved CK1 regions and phylogenetic analysis. **(A)** Multiple sequence alignment of the *Leishmania* CK1s (**Table 1**) was generated using the T-COFFEE Server (http://tcoffee.crg.cat (Notredame et al., 2000)). The dark blue corresponds to the N- and C-termini; the lighter blue corresponds to the kinase domain; and the lightest blue corresponds to insertions in the catalytic domain. The conserved amino acids were visualized by Jalview; the color code is as follows, low level of amino acid conservation (dark brown) to highly level of conservation (yellow) (https://www.jalview.org (Waterhouse et al., 2009)). **(B)** Schematic representation of the domain organisation of the different *Leishmania* CK1s, see A for the color code. **(C)** Phylogenetic tree of all the trypanosomatid CK1s described in the text (see **Table 1**) obtained using the http://www.phylogeny.fr server (Dereeper et al., 2008). The bar represents an evolutionary distance of 0,3%.

The *Leishmania major* CK1 family consists of 6 paralogs (**Table 1** (Dan-Goor et al., 2013)). The LmaCK1.1 and LmaCK1.2 genes are adjacent on chromosome 35 and closely related, suggesting that they have a common ancestor (Rachidi et al., 2014). In contrast, the four others paralogs (LmaCK1.3-6) are quite divergent from LmaCK1.1 and LmaCK1.2, with for instance amino acids insertions observed in the kinase domain of LmaCK1.4 and LmaCK1.5 (**Figures 1A, B** (Rachidi et al., 2014)). Although little is known about their functions, recent work by Baker et al. showed in *L. mexicana* that only LmxCK1.2 and LmxCK1.4 are essential, which incidentally are the only two paralogs being excreted by the parasite (Silverman et al., 2010; Dan-Goor et al., 2013; Baker et al., 2020). This was pharmacologically confirmed for *L. donovani* LdCK1.2, indicating that CK1.2 could be essential across all *Leishmania* species (Rachidi et al., 2014; Durieu et al., 2016).

**Table 1:** CK1 in parasites.

Organisms	Name	Identifiers	% to *L. major* ortholog	MW in kDa	Phosphorylated sites	% Identity to human CK1 (% coverage)
***Plasmodium falciparum*** ***3D7***	PfCK1	PF3D7_1136500	62% LmaCK1.2	38	S17, T313	69% human CK1δ (92%)
***Toxoplasma gondii*** **ME49**	TgCK1α	TgME49_240640	69% LmaCK1.2	38	ND	73% human CK1δ (92%)
	TgCK1β	TgME49_289320	59% LmaCK1.2	49	S38, S335, T338, S372, T375, S389	65% human CK1δ (69%)
	TgCK1γ	TgME49_247710	53% LmaCK1.2	61	ND	65% human CK1δ (88%)
***Crypto-*** ***sporidium parvum***	CpCK1α	XP_001388103.1	67% LmaCK1.2	48	ND	72% human CK1δ (68%)
	CpCK1β	XP_001388088.1	60% LmaCK1.2	56	ND	72% human CK1δ (68%)
***Trypanosoma brucei*** ***TREU927***	TbCK1.1	Tb927.5.790	73% LmaCK1.2 63% LmaCK1.1	38	S326, T329	65% human CK1δ (88%)
	TbCK1.2	Tb927.5.800	88% LmaCK1.2	38	S21, S324	69% human CK1δ (89%)
	TbCK1.3	Tb927.10.2390	49% LmaCK1.3	42	ND	38% human CK1δ (75%)
	TbCK1.3a	Tb11.v5.0577	49% LmaCK1.3	42	ND	38% human CK1δ (75%)
	TbCK1.5	Tb927.3.1630	42% LmaCK1.5	45	S7, S12, S13, S15, S25, S29	47% human CK1α (80%)
***Trypanosoma cruzi*** ***DM28c 2018***	TcCK1.1	C4B63_111g31	80% LmaCK1.2 65% LmaCK1.1	36	ND	67% CK1ϵ (94%)
	TcCK1.2	C4B63_111g32 to C4B63_111g56, C4B63_111g124c and C4B63_111g154c, C4B63_15g1 to C4B63_15g3, C4B63_397g3 to **C4B63_397g10**	**84% LmaCK1.2**	**38**	**ND**	**70% human CK1δ (89%)**
	TcCK1.3	C4B63_79g2	61% LmaCK1.3	42	ND	42% human CK1α2 (79%)
	TcCK1.3a	C4B63_312g11	65% LmaCK1.3	29	ND	45% human CK1α (90%)
	TcCK1.5	C4B63_11g86	45% LmaCK1.5	47	S118, S120, S361	46% human CK1δ (85%)
	TcCK1.6	C4B63_18g160	57% LmaCK1.6	44	ND	48% human CK1δ (74%)
***Leishmania major*** ***Friedlin***	LmaCK1.1	LmjF.35.1000	N/A	37	ND	61% human CK1δ (89%)
	LmaCK1.2	LmjF.35.1010	N/A	40	S19, S21	69% human CK1δ (83%)
	LmaCK1.3	LmjF.04.1210	N/A	41	ND	36% human CK1α (87%)
	LmaCK1.4	LmjF.27.1780	N/A	61	ND	31% human CK1α (57%)
	LmaCK1.5	LmjF.25.1580	N/A	58	ND	48% human CK1δ (63%)
	LmaCK1.6	LmjF.30.3470	N/A	42	ND	49% human CK1δ (77%)
***Giardia intestinalis***	GiCK1.2	ESU42061.1	58% LmaCK1.2	47	ND	66% human CK1ϵ (72%)
***Entamoeba histolytica*** ***HM-1:IMSS***	EhCK1.2a	XP_657192.1	55% LmaCK1.2	36	ND	58% human CK1α (98%)
	EhCK1.2b	XP_655864.1	51% LmaCK1.2	35	ND	52% human CK1δ (98%)
	EhCK1.2c	XP_652927.1	52% LmaCK1.2	36	ND	54% human CK1α (97%)
	EhCK1.2d	XP_657385.1	53% LmaCK1.2	39	ND	57% human CK1ϵ (88%)
	EhCK1.2e	XP_650630.1	44% LmaCK1.2	33	ND	50% human CK1δ (94%)

Bold: For T.Cruzi the data presented in the table are only for C4B63_397g10.

Casein kinase 1 (CK1), amino acids (aa), Molecular Weight (MW).

#### Leishmania CK1.2

CK1.2 is the most conserved kinase among *Leishmania* species, unlike CK1.1, and has the highest identity to its human orthologs, with the closest human orthologs being CK1 δ and ϵ (Xu et al., 2019). This conservation provides evidence that the CK1.2 protein sequence may be under evolutionary pressure to resemble that of host CK1s (Rachidi et al., 2014), either to insure recognition of the host CK1 substrates by released *Leishmania* CK1.2, to avoid recognition of L-CK1.2 by the immune system or both. CK1.2 has nevertheless two unique characteristics compared to its human orthologs. First, the insertion of a GGA sequence between domain III and IV in the kinase domain, which is specific to the trypanosomatids and can be exploited for the development of specific inhibitors (see dedicated chapter below) (Spadafora et al., 2002; Rachidi et al., 2014). The significance of this insertion is still unknown. Second, parasite-specific N- and C-termini, which seem to regulate LdCK1.2 localisation and abundance. The C-terminus contains two low complexity regions (LCRs) that might be important for its subcellular localisation while its N-terminus seems essential to maintain a detectable level of LdCK1.2 in the parasite (Knippschild et al., 2014; Martel et al., 2020).

LdCK1.2 was shown to be one of the major kinase activities in *Leishmania*, representing on its own 46% of the total kinase activity in promastigotes and 58% in axenic amastigote (Rachidi et al., 2014). As such, this kinase needs to be tightly regulated. However, unlike human CK1α, δ and ϵ that are auto-inhibited by auto-phosphorylation (Knippschild et al., 2014), L-CK1.2 is constitutively active and thus requires alternative mechanisms of regulation. One major mechanism is the regulation of L-CK1.2 enzymatic activity by temperature. Indeed, LdCK1.2 activity decreases by half when promastigotes (26˚C) differentiate into axenic amastigotes (37˚C) (Rachidi et al., 2014). Although the mechanisms leading to this decrease are unknown, recent evidence suggests two possibilities: First, the decrease in activity could be the consequence of serine S19 and S21 phosphorylation, which are part of the ATP binding site and exclusively modified in axenic amastigotes (Tsigankov et al., 2013). Second, LdCK1.2 activity could be regulated by heat shock proteins, given that LdCK1.2 phosphorylates the two chaperones Hsp70 and Hsp90, and the two co-chaperones Hsp23 and P23. Indeed, CK1.2 was shown to co-localise with Hsp70 to many organelles, such as flagellar tip or basal body, suggesting a role of this chaperone in the regulation of CK1.2 localisation rather than regulation of its activity (Xu et al., 2019; Martel et al., 2020). LdCK1.2 also co-localises with Hsp90 to the flagellar pocket neck and phosphorylates the chaperone at serine S289, which is situated in the client protein recognition domain (**Table 2** (Hombach-Barrigah et al., 2019; Martel et al., 2020)). Abrogation of this phosphorylation in promastigotes leads to a growth defect, decrease in flagellum length, and reduced infectivity, suggesting that Hsp90 regulation by LdCK1.2 is important for parasite biology and virulence (Hombach-Barrigah et al., 2019). Thus, LdCK1.2 seems to regulate Hsp90 rather than the opposite. Finally, recent data have demonstrated a genetic link between LdCK1.2 and Hsp23, a co-chaperone that regulates Hsp90 activity (Kröber-Boncardo et al., 2020). The loss of viability of Hsp23-deletion mutants at high temperature was rescued by overexpression of LdCK1.2 (Kröber-Boncardo et al., 2020). Moreover, Hsp23 seems to have an impact on LdCK1.2 activity, reducing its affinity for substrates and increasing auto-phosphorylation (Kröber-Boncardo et al., 2020). In absence of Hsp23, overexpression of LdCK1.2 might rescue Hsp90 deregulation by increasing its phosphorylation (Kröber-Boncardo et al., 2020). Further investigation will be required to test this possibility and confirm whether Hsp23 regulates LdCK1.2 activity *in vivo*.

**Table 2:** Parasite and host substrates of parasitic CK1s.

Parasites CK1	Parasite substrates		
*Plasmodium falciparum* PfCK1	Name/phosphosite	Function	References
	PfRON3	Rhoptry protein	(Dorin-Semblat et al., 2015)
	PfNapL	Nucleosome assembly protein	(Dorin-Semblat et al., 2015)
	PfAlba4	DNA/RNA-binding protein	(Dorin-Semblat et al., 2015)
*Trypanosoma brucei* TbCK1.2	ZC3M11	RNA-binding protein	(Minia and Clayton, 2016)
*Leishmania donovani* LdCK1.2	Hsp90 Serine 289	Chaperone protein	(Hombach-Barrigah et al., 2019)
	Hsp70	Chaperone protein	(Martel et al., 2020)
	Hsp23	Chaperone protein	(Kröber-Boncardo et al., 2020)
	P23	Co-chaperone protein	(Kröber-Boncardo et al., 2020)
**Parasite** **CK1**	**Host substrates**		
	Name/phosphosite	Function	Reference
*Leishmania* *major* LmaCK1.2	IFNAR1 Serine 535	Subunit of the heterodimeric receptor complex for type 1 interferon signals	(Liu et al., 2009)

LdCK1.2 is also regulated by more conventional mechanisms similarly to its mammalian orthologs, such as subcellular sequestration. LdCK1.2 localises to the flagellum, the flagellar pocket, the granular zone of the nucleolus, the basal body and the mitotic spindle, which suggests roles in parasite motility, in ribosome biogenesis, or in kinetoplast and chromosome segregation (Martel et al., 2020). Further investigations exploring interacting partners will allow deciphering *Leishmania* CK1.2 functions.

#### Leishmania CK1.4

The second essential CK1 is LdCK1.4. Absent in the *Trypanosoma* spp. genome, LdCK1.4 shows only 31% identity to human CK1α, its closest human ortholog (Baker et al., 2020). This unusual CK1 has a long N-terminal domain and large insertions in its kinase domain, which do not affect its enzymatic activity, despite potentially affecting kinase structure. In contrast, deleting both the N- and C-termini of LdCK1.4 abrogates kinase activity (Dan-Goor et al., 2013). In that respect, LdCK1.4 differs from LdCK1.2, as deletion of its N- and C-termini does not affect enzymatic activity (Martel et al., 2020). LdCK1.4 localises to the cytoplasm and the flagellar pocket (Dan-Goor et al., 2013; Baker et al., 2020; Martel et al., 2020). Overexpression of LdCK1.4 leads to an increase in metacyclic parasite concentration and an increase in the percentage of infected macrophages, suggesting an involvement in metacyclogenesis and infectivity. Because of the recent discovery of its essential nature in parasite viability, it would be important to investigate this paralog further.

#### Other CK1 Paralogs

The four remaining paralogs are dispensable for *Leishmania mexicana* survival in *Leishmania mexicana* (Martel et al., 2017; Baker et al., 2020), suggesting that they are either redundant, or regulate none-essential functions. Data are mostly available for CK1.1. Martel et al. showed that CK1.1 is dispensable for *Leishmania donovani* promastigote and axenic amastigote survival or axenic differentiation, but may be involved in the regulation of stationary phase processes (Martel et al., 2017). Likewise, *L. mexicana* LmxCK1.1 is dispensable for parasite survival in the sand fly in the mammalian host (Baker et al., 2020; Martel et al., 2020). Apart from the transcriptomic and proteomic data available in TriTrypDB (https://tritrypdb.org/tritrypdb/app), only localisation data are available on the three, showing that LmxCK1.3 and LmxCK1.6 display a cytoplasmic and CK1.5 a flagellar localization (Aslett et al., 2010; Fernandes et al., 2016).

### Casein Kinase 1 Family Members in Trypanosoma

CK1 family members are also present in *Trypanosoma brucei* and *Trypanosoma cruzi*. Although closely related to those of *Leishmania* (**Figure 1C**), they are different in terms of genomic organisation. First, the number of paralogs varies, from six in *Leishmania* spp. to five in *T. brucei* TREU 927 (**Table 1**) and four in *T. cruzi* DM28c 2018 (**Table 1**). Notably, the two essential kinases CK1.2 and CK1.5, as well as non-essential CK1.3 are conserved. Second, the gene copy number of the different paralogs varies according to the genus, which is well illustrated by CK1.2. Whereas a single gene copy encodes for this kinase in *Leishmania* spp. and in *Trypanosoma brucei*, it is organized as a multigenic family in *Trypanosoma cruzi* DM28c 2018, with 32 genes and 7 gene fragments spread over three clusters of tandem repeats (**Table 1** (Aslett et al., 2010)). Furthermore, the copy number shows strain-specific variations: *T. cruzi TCC* has 49 genes in 12 tandem gene clusters, whereas *T. cruzi CL Brener Non-Esmeraldo-like* has only 4 genes in one cluster (Aslett et al., 2010). It would be important to determine whether a high number of isoforms contributes to parasite survival, although it seems unlikely considering that *T. cruzi CL Brener Non-Esmeraldo-like* has only 4 copies. The difference in CK1.2 gene copies between species might rather be due to retrotransposon copy number variations between strains, as they contribute to genomic plasticity (Berna et al., 2018), a phenomenon also described for *Saccharomyces cerevisiae* (Rachidi et al., 1999). Indeed, TCC, which has the highest number of CK1.2 genes has also the highest number of retrotransposons. The biological function of CK1.2 gene amplification is unknown, but in *Leishmania* it is usually associated with an increase in transcript abundance, as a way to compensate for the absence of transcriptional regulation (Prieto Barja et al., 2017). Thus, for some *T. cruzi* species it might be crucial to express high levels of TcCK1.2, as a way to adapt to environmental changes. In that respect, the genomic organisation of TcCK1.2 resembles that of HSPA1 and HSPA9, encoding *Leishmania* HSP70 chaperone proteins, whose copy number vary between strains (Drini et al., 2016). Further investigations will be required to determine whether the amplification of TcCK1.2 corresponds to an adaptive mechanism.

The conservation and expansion of CK1.2 in *Trypanosoma* suggest important functions. Indeed, CK1.2 is essential in *T. brucei* and its depletion results in an increase in 2N2K cells, pointing to a cell cycle arrest in mitosis, and an increase of abnormal cells, mostly 2N1K or aneuploid cells (Urbaniak, 2009; Jones et al., 2014). TbCK1.2 might thus have a role in kinetoplast division as well as cytokinesis, which is consistent with the localisation of its *Leishmania* ortholog LdCK1.2 to the basal body and the flagellar pocket (Knippschild et al., 2014; Jones et al., 2014; Martel et al., 2020). TbCK1.2 also acts as a negative regulator of ZC3H11 (**Table 2**), a CCCH zinc finger protein required to stabilize stress response mRNAs upon heat shock (Minia and Clayton, 2016). Thus TbCK1.2 may be a negative regulator of mRNA stability. At 27˚C, TbCK1.2 is active and phosphorylates ZC3H11, leading to its destabilisation; upon heat shock (41°C) TbCK1.2 becomes inactive, ZC3H11 is no longer phosphorylated and thus accumulates to stabilize stress response mRNAs. The inactivation of TbCK1.2 at 41°C is consistent with the decrease of CK1.2 activity at 37°C observed in *Leishmania* (Rachidi et al., 2014), supporting the hypothesis that CK1.2 orthologs might be temperature sensitive enzymes.

## CK1 in Apicomplexa

In contrast to trypanosomatids, the genomes of apicomplexan parasites of the genus *Plasmodium* and *Toxoplasma* only encode for orthologs of CK1.2, defining this CK1 family member as a key kinase across all major human parasites.

The *Plasmodium* genome encodes for a single member of the CK1 family (PfCK1, **Table 1** (Ward et al., 2004)), which is highly unusual compared to other eukaryotes such as trypanosomatids (see above), *S. pombe* (2 paralogs) or *C. elegans* (85 paralogs). PfCK1 is regulated at three levels: (i) at transcript level, being expressed as two alternative transcripts of 2,4 and 3,2 kb with highest abundance observed during the early erythrocytic ring stage (Barik et al., 1997); (ii) at protein level, given that stage-specific expression of the 37-kDa protein does not correlate to transcript levels (Dorin-Semblat et al., 2015); and (iii) at post-translational level, as CK1 phosphotransferase activity is higher at ring stage compared to trophozoites, while being almost undetectable in schizonts (Barik et al., 1997). The enzymatic activity of PfCK1 could be regulated through the phosphorylation of Ser17, Ser19, or T313 (Aurrecoechea et al., 2009; Treeck et al., 2011; Lasonder et al., 2012a; Lasonder et al., 2012b; Pease et al., 2013). Indeed, the phosphorylation of S17 in the ATP binding site, peaks at ring stage, similarly to the kinase activity (Pease et al., 2013). In that respect, PfCK1 displays similar regulation as trypanosomatid CK1.2. Based on recent data, PfCK1 interacts with proteins involved in various pathways, some being also substrates (**Table 2**). Enrichment of the PfCK1 interactome in biological processes such as post-translational modification, transcription, translation, and protein trafficking (**Tables 2** and **3** (Dorin-Semblat et al., 2015)), reveals pleiotropic functions of this kinase. For instance, the interaction of PfRON3 (RhOptry Neck protein 3) with PfCK1 and its phosphorylation might have a role in invasion. The two proteins partially co-localise in early schizonts, while PfCK1 is associated with micronemes in merozoites (Dorin-Semblat et al., 2015).

**Table 3:** Parasitic and host binding partners of parasitic CK1s.

Parasites CK1	Parasite binding partners		
*Plasmodium falciparum* PfCK1	Name	Functions	References
	PfCK2α	Casein kinase 2 α protein kinase	(Dorin-Semblat et al., 2015)
	PfCK2β	Casein kinase 2 β protein kinase	(Dorin-Semblat et al., 2015)
	PfRON3	Rhoptry protein	(Dorin-Semblat et al., 2015)
	PfNapL	Nucleosome assembly protein	(Dorin-Semblat et al., 2015)
	PfRab5B	GTPase guanine nucleotide-binding protein	(Rached et al., 2012)
**Parasite** **CK1**	**Host binding partners**		
*Plasmodium* *falciparum* PfCK1	Name	Functions	Reference
	GAPVD1	GTPase-activating protein and VPS9 domain-containing protein 1	(Batty et al., 2020)

In contrast to *Plasmodium*, *Toxoplasma gondii* has three CK1.2 isoforms: TgCK1α, TgCK1β and TgCK1γ, with respectively 78%, 63% and 59% identity to PfCK1 (**Table 1**, Gajria et al., 2008). The different length of their N- or C-terminal domains points to specific regulations or localisations. Indeed, TgCK1β is phosphorylated at S38 (ATP-binding site), S335, T338, S372 T375 and S389, while TgCK1α does not seem to be phosphorylated (Treeck et al., 2011). TgCK1β is localised at the plasma membrane, while TgCK1α is detected in extracellular vesicles as observed for LdCK1.2 (Donald et al., 2005). TgCK1α was shown to be non-essential in *T. gondii GT-1* by Wang et al. (2016). The knockout mutant showed a delay in intracellular parasite proliferation, but was normal in host cell attachment and invasion. This data is in contradiction with Sidik et al. Applying a CRISPR/Cas9 screen on the same strain (Sidik et al., 2016), they provided evidence that TgCK1α is essential (in contrast to TgCK1β and TgCK1γ), since its deletion leads to a sharp decrease in intracellular parasite fitness (Sidik et al., 2016). The discrepancies between these two studies might be explained by the different host cells used (African green monkey kidney (Vero) (Wang et al., 2016) versus human fibroblast (HFF) (Sidik et al., 2016)). Nevertheless, an essential function of TgCK1α would be consistent with its orthologs PfCK1 (Solyakov et al., 2011), TbCK1.2 (Urbaniak, 2009) and *Leishmania* CK1.2 (Rachidi et al., 2014; Baker et al., 2020).

## Roles of Parasitic CK1 in Host-Pathogen Interactions

Successful pathogens manipulate their host through the release of pathogen-derived molecules and direct interaction with host signaling proteins (Nandan et al., 2002; Olivier et al., 2005; Silverman et al., 2010; Dorin-Semblat et al., 2015). The secretion of molecules into host cells as a way to establish permissive conditions for intracellular survival has been shown for many parasites (for review see (Ofir-Birin and Regev-Rudzki, 2019)). Among these molecules, increasing evidence suggests that excreted CK1 could be a key regulator of host-pathogen interactions, acting as a trans-signaling kinase *via* phosphorylation of host substrates.

Notably, while the paralogs CK1.2, CK1.4 and CK1.5 seem to have extra-parasitic role in *Leishmania*, only orthologs of CK1.2, namely PfCK1 and TgCK1α, have been shown to be released in other parasites.

### CK1.2 Orthologs Are Key Enzymes in Host-Parasite Interaction

Aside from its functions inside the parasite, *Leishmania* CK1.2 is secreted, and excreted *via* exosomes (Sacerdoti-Sierra and Jaffe, 1997; Vieira et al., 2002; Silverman et al., 2008; Silverman et al., 2010; Dan-Goor et al., 2013; Atayde et al., 2015). Leishmania exosomes were shown to attenuate the immune response of the host cell to favor parasite survival (Silverman et al., 2011). They are also co-inoculated with the parasites to the mammalian host by blood feeding sand flies, and shown to exacerbate the disease (Atayde et al., 2015). Moreover, recent data have shown that exosomes might play a role in parasite chemio-resistance, as exosomal cargo adapts to drugs. For instance, exosomal *Leishmania* CK1.2 is enriched in amphotericin B-resistant parasites but not in miltefosine- or antimony-resistant parasites (Douanne et al., 2020). The identification of *Leishmania* CK1.2 in EVs suggests a role for the parasite kinase in the host cell. Indeed, *Leishmania* CK1.2 phosphorylates the destruction motif (degron) of the human IFNAR1 receptor on serine S535, which allows the recruitment of an E3 ubiquitin ligase to facilitate its ubiquitination, endocytosis and subsequent degradation (Liu et al., 2009). IFNAR1 removal from the plasma membrane leads to attenuation of the cellular response to type 1 interferon α/β *in vitro* (Liu et al., 2009). Because IFNAR1 is also a substrate of human CK1α, this finding demonstrates the ability of *Leishmania* CK1.2 to phosphorylate host CK1 substrates and to attenuate the immune response. Considering the pleiotropic role of human CK1s in processes such as apoptosis or protein transport (Xu et al., 2019), many more host pathways could be regulated by LdCK1.2 to manipulate the biological and immune functions of the host macrophage.

TgCK1α has also been detected in extracellular vesicles (Donald et al., 2005) but the role of the excreted kinase has not yet been investigated. Nevertheless, TgCK1α seems to be indirectly required to limit the pathogenicity of the parasite. Indeed, the deletion of TgCK1α increases clinical signs and acute virulence in mice by increasing the transcription of ROP (Rhoptry) genes such as ROP16, which has been shown to phosphorylate STAT3 and thus to inhibit IL-12 production (Wang et al., 2016). Disease exacerbation in TgCK1α deletion mutants eliminates this kinase as a potential drug target.

Finally, the *Plasmodium* CK1.2 ortholog PfCK1 is also excreted into its erythrocytic host cell. PfCK1 is associated with the RBC cell surface during the early stages of infection (Florens et al., 2004; Dorin-Semblat et al., 2015) and released from infected RBCs at the early trophozoite stage, suggesting that it might be implicated in the modification of bystander RBCs or epithelial cells (Dorin-Semblat et al., 2015). This hypothesis is consistent with the finding that purvalanol B – a major interactor of PfCK1- kills *Plasmodium* parasites despite its poor membrane permeability potentially by targeting the extracellular fraction of PfCK1 (Knockaert et al., 2000; Dorin-Semblat et al., 2015). More recently, Batty et al. have identified host proteins involved in membrane trafficking that interact with PfCK1 at the trophozoite stage, including GTPase Activating Protein and VPS9 Domains 1(GAPVD1) and Sorting Nexin 22 (SNX22) (Batty et al., 2020). These interactions might contribute to the trafficking of PfCK1 to the cell surface (Dorin-Semblat et al., 2015; Batty et al., 2020). The mechanisms of PfCK1 export remains elusive, as the kinase does not (i) contain a PEXEL export motif (Plasmodium EXport ELement), (ii) interact with the Maurer’s clefts or (iii) localise to extracellular vesicles (Abdi et al., 2017; Batty et al., 2020). There is some evidence that it might be released by rhoptries through interaction with ROP3, and/or micronemes (Florens et al., 2004; Dorin-Semblat et al., 2015).

### Potential Extracellular Functions of CK1.4 and CK1.5

LdCK1.4 is secreted in the supernatant of cultured *L. donovani* promastigotes, and although a signal peptide, required for non-classical and classical protein secretion, has been found using dedicated software, it has not been confirmed *in vivo* (Dan-Goor et al., 2013). LinCK1.4 was also identified in the proteome of exosomes collected from logarithmic but not from stationary phase *L. infantum chagasi* promastigotes (Forrest et al., 2020) but not in any other exoproteomes (Silverman et al., 2010; Silverman et al., 2011; Atayde et al., 2015; Douanne et al., 2020). Thus, the presence of CK1.4 in exosomes will have to be confirmed by further proteomics studies. Due to its weak identity to its human orthologs and the divergent structure of its kinase domain (**Figures 1A, B**), it seems unlikely that CK1.4 would target human CK1 substrates; it may rather target *Leishmania* extracellular proteins or *Leishmania*-specific human substrates.

CK1.5 has been recently identified in exosomes released by antimony-resistant *Leishmania*, suggesting that excreted CK1.5 could be important for parasite resistance (Douanne et al., 2020).

### Structural and Functional Comparison of Human CK1 and Parasite CK1 Orthologs

**Figure 2:**
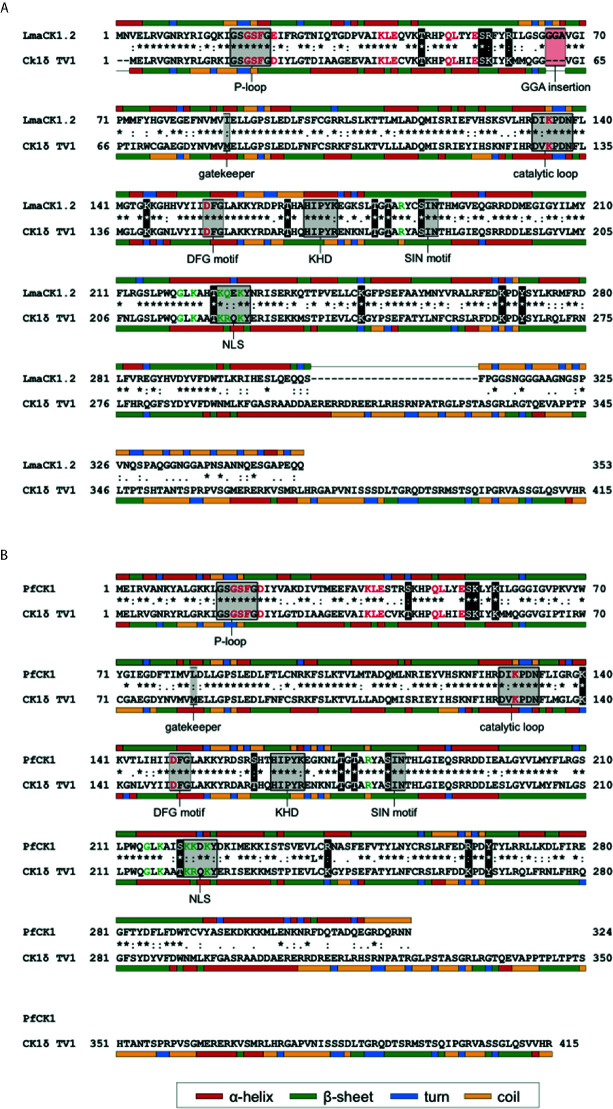
Sequence alignment of LmaCK1.2 **(A)** and PfCK1 **(B)** with human CK1δ TV1. **(A)** Protein sequences of the major casein kinase 1 (CK1) isoform of *Leishmania* (LmaCK1.2) and human CK1δ TV1 were aligned using ClustalW (Thompson et al., 1994). Full conservation of amino acids (*), strong conservation (:) and weak conservation (.) is indicated in the line between the sequences. Secondary structure features were predicted using CFSSP (Ashok Kumar, 2013) and are indicated by a color code next to the respective sequence. Specific motifs, residues involved in ATP-binding (red characters), as well as residues involved in substrate recognition (green characters) for human CK1δ are indicated in the figure as well as the alternative residue in LmaCK1.2, if different. **(B)** Protein sequences of PfCK1 of *Leishmania* (LmaCK1.2) and human CK1δ TV1 were aligned as described in **(A)**.

CK1.4 and CK1.5 release in the host cell seems specific to *Leishmania*, as it has not been shown for any orthologs in other parasites in contrast to CK1.2. One explanation might be that CK1.2 is particularly important as it can substitute for host CK1s during parasite infection. Several findings support this hypothesis. First, *Leishmania* CK1.2 is the most closely related kinase to human CK1δ, CK1ϵ and CK1α and might be evolutionary selected for its interaction with host substrates. Second, Isnard et al. have shown that in the nuclear fraction of *L. major*- and *L. mexicana*-infected murine macrophages, the level of host cell CK1α is decreased compared to non-infected macrophages (Isnard et al., 2015), suggesting that *Leishmania* CK1.2 might substitute for the host kinase. Notably, CK1α is the host isoform that phosphorylates IFNAR1 (Liu et al., 2009). Third, most common structural features and regulatory residues in human CK1s are present in LmaCK1.2 and PfCK1 (**Figure 2**) (Xu et al., 2019). Due to the pleiotropic processes regulated by mammalian CK1 family members, these kinases need to be tightly regulated. Post-translational modifications, such as acetylation, ubiquitination, neddylation, or phosphorylation, have been identified to play an important role in regulating host CK1 functions. Indeed, phosphorylation of human CK1δ within its kinase domain and its C-terminal domain by PKC, PKA, CDKs, or Chk1, results in changes of CK1δ activity (Giamas et al., 2007; Bischof et al., 2013; Ianes et al., 2016; Meng et al., 2016; Eng et al., 2017; Böhm et al., 2019). Several authors have shown that within the kinase domain of CK1δ, Chk1 phosphorylates residues T161, T174, T176, S181 and T220, whereas PKCα targets S53, T176, and S181. Considering that LmaCK1.2 and PfCK1 may assume host CK1 functions, they could be regulated similarly. An interspecies comparison focusing on LmaCK1.2 and PfCK1 revealed that regulatory residues of host CK1δ (T161, T174, T176 and S181) were conserved in the parasite orthologs indicating that their phosphorylation status could be important for the regulation of parasite CK1-specific activity and/or functions in their respective host cells (**Figure 2** (Böhm et al., 2019)). T220 is conserved in LmaCK1.2 but not completely in PfCK1 where it is substituted by serine (**Figure 2** (Böhm et al., 2019)). Moreover, S53, which is specifically targeted by PKCα, is also conserved in PfCK1 and LmaCK1.2 (**Figure 2** (Meng et al., 2019)). Although conserved, these sites are neither phosphorylated in *Leishmania* nor in *Plasmodium* (Tritrypdb, Plasmodb). These findings suggest the intriguing possibility that they might only be phosphorylated after release into the host cell. In contrast, only human CK1ϵ has been shown to be phosphorylated at serine S17 within the conserved glycine-rich ATP binding loop (p-loop) similarly to PfCK1 and LmaCK1.2 (Nett et al., 2009; Treeck et al., 2011; Tsigankov et al., 2013). Moreover, the lysine residues that are ubiquitinated or acetylated in CK1δ and CK1ϵ are also conserved in PfCK1 and LmaCK1.2. Comparing only the kinase domain (given the divergence of the N- and C-termini), the organization of kinase-specific sub-domains and the regulatory sites of human CK1δ, ϵ are conserved in PfCK1 and LmaCK1.2, supporting the hypothesis that the two parasite kinases can substitute for human CK1s. Despite these similarities, the parasite kinases still retain specific features such as amino acid changes as well as the GGA amino acid insertion between sub-domains III and IV forming the ATP binding pocket, that on one hand could influence CK1 activity, regulation and functions and on the other hand may be exploited to develop parasite CK1-specific inhibitors (Rachidi et al., 2014).

## CK1 as Drug Target

The essential nature of CK1 family members in most parasites defines these kinases as important target candidates for anti-parasitic therapy (Urbaniak, 2009; Solyakov et al., 2011; Rachidi et al., 2014). The dual functions of some of these kinases both in parasite biology and subversion of the host cell, likely increases the genetic barrier for the development of drug resistant parasites (Lamotte et al., 2017). To date, only *Leishmania* CK1.2 was used in drug screenings (Allocco et al., 2006; Marhadour et al., 2012; Marchand et al., 2015; Durieu et al., 2016; Bazin et al., 2020). Although L-CK1.2 is closely related to human CK1s (about 59%), these drug screens demonstrated that small molecules could easily discriminate between the two kinases. Indeed, the position of F22 and K40, two residues present in the active site of L-CK1.2, might change its shape and explain the difference in affinity of the two kinases toward the same compound (Durieu et al., 2016). Moreover, in the kinase domain, trypanosomatid CK1s display an insertion of three amino acids that might also contribute to this discrimination (Rachidi et al., 2014). Mutagenesis should be performed in order to confirm this hypothesis. To improve the identification of novel and specific L-CK1.2 inhibitors, Durieu et al. have developed a drug discovery pipeline that employs a dual screen against the recombinant parasite and mammalian CK1, tests parasite-specific hits for anti-leishmanial activity in culture and infected macrophages, and assesses compound cytotoxicity and its affinity to L-CK1.2 (Durieu et al., 2016). Using this pipeline, thousands of compounds were screened and two hit compounds with selectivity index above 10 were identified (Durieu et al., 2016). The potential functions of L-CK1.2 in infected macrophages may render this target more refractory to drug resistance, as any CK1.2 mutation that avoids inhibitor binding might compromised its extra-parasitic functions and ultimately intracellular parasite survival (Rachidi et al., 2014). However, the main challenge in targeting parasite orthologs of human CK1s lies in gaining drug selectivity and avoiding cell toxicity, although this is less of a concern for *Leishmania* CK1.2 given its structural differences compared to the human ortholog in the ATP-binding site. However, as shown by Rachidi et al., such toxic compounds could be eliminated very early in the drug discovery process by comparative screening on both recombinant mammalian and parasitic kinases (Durieu et al., 2016). Altogether, these findings define *Leishmania* CK1.2 as an important drug target, and given its similarities to the orthologs of the pathogenic parasites described in **Table 1**, CK1.2 paralogs have the potential of becoming a pan-parasitic drug targets.

## Conclusions

**Figure 3:**
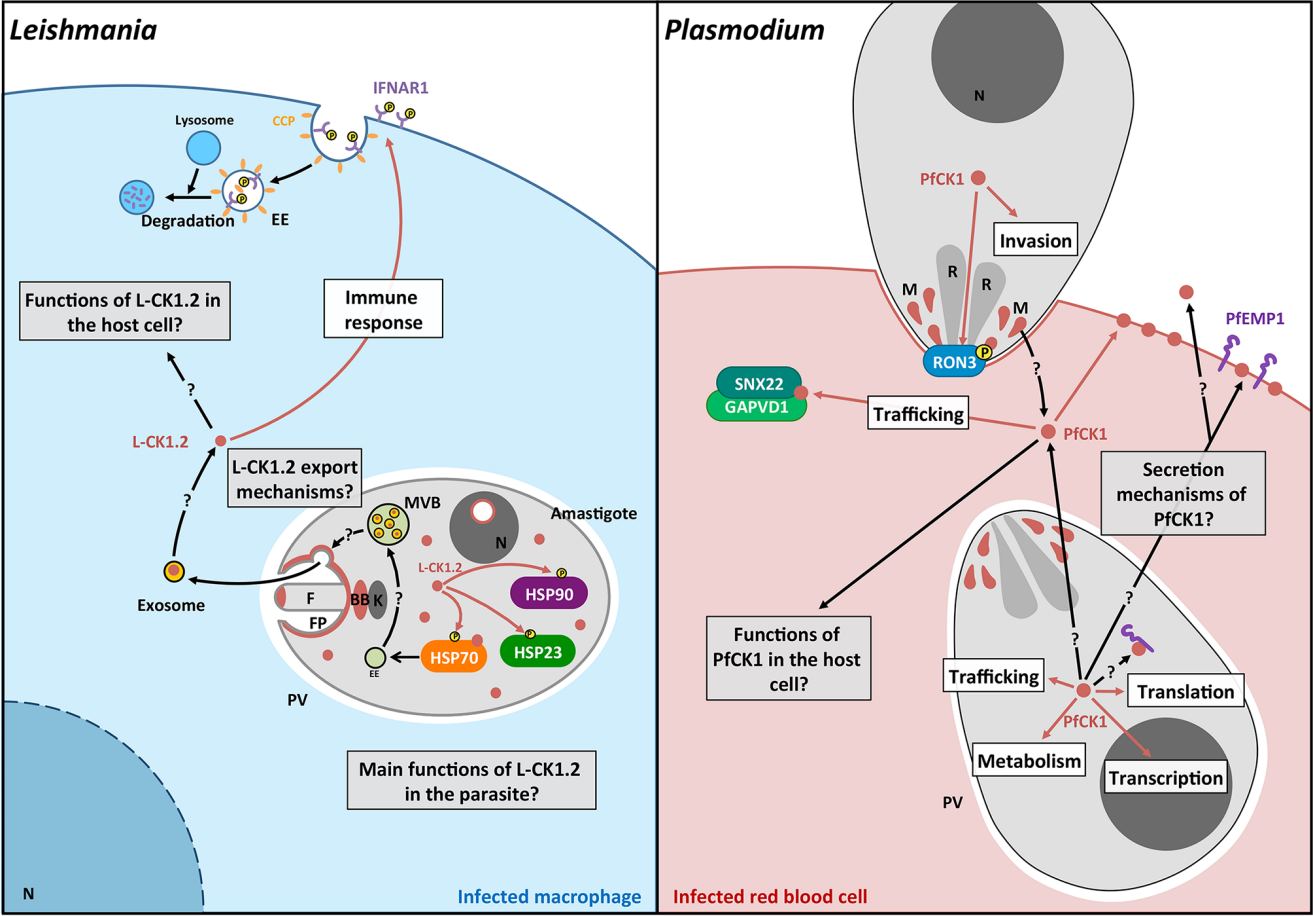
Schematic representation of the pathways regulated by L-CK1.2 and PfCK1. **Left panel.** L-CK1.2 is localised in the parasite at the flagellar tip, the flagellar pocket, the basal body, the nucleolus and the cytoplasm as shown in brown. It phosphorylates three heat shock proteins as symbolized by brown arrows. L-CK1.2 has been identified in exosomes although the mechanisms leading to its loading into exosomes and its release are currently unknown. L-CK1.2 has functions in the macrophage, particularly in innate immunity (white box), however, most of its functions remain to be identified (grey box). **Right panel.** PfCK1 seems to be important for invasion either through phosphorylation of Ron3 or release by micronemes. In the parasite, PfCK1 seems to be involved in functions such as translation or trafficking (white box) but the specific mechanisms need to be elucidated. PfCK1 seems to be also released in red blood cells (RBC) as well as in the extracellular environment by unknown mechanisms. One function of PfCK1 in RBCs could be related to the regulation of trafficking through the binding of two host proteins. White boxes correspond to potential functions; Grey boxes correspond to key questions; Brown arrows correspond to known mechanisms. Black arrows correspond to mechanisms that need to be identified. F, Flagellum; FP, Flagellar pocket; BB, Basal body; k, kinetoplast; EE, Early endosome; MVB, Multivesicular bodies; N, Nucleus; CCP, Clathrin coated pit; P, phosphorylation; R, Rhoptry; M, Micronemes.

This review documents the importance of the CK1 family in the biology of all major human protozoan parasites, and its crucial roles in regulating host-pathogen interactions during *Leishmania*, *Plasmodium* and *Toxoplasma* infection (**Figure 3**). However, many aspects of parasitic CK1s are still unknown (**Figure 3**). Future research will have (i) to expand knowledge on their biological functions in the parasite for instance by screening for their binding partners and substrates (**Tables 2** and **3**); (ii) to elucidate the mechanism of CK1 release into the host environment, as well as their role in immune and metabolic host subversion; and (iii) to determine mechanisms underlying CK1-mediated and infection-related aspects of host-parasite interactions, both for intra- and extracellular parasites. Knowing how parasite CK1s contribute to the subversion of host cell signaling will be crucial to understand host-parasite interactions and to find new therapies. Indeed, blocking the release of excreted CK1s or interfering with its extra-parasitic functions to restore the host cells’ biological capabilities would be an efficient way to kill the parasite. Combining functional genetics and phospho-proteomics, future analyses will confirm CK1 as an essential kinase family in host-pathogen interactions, thereby paving the way to exploit other trans-signaling proteins for host-directed drug discovery.

## Author Contributions

All authors have been involved in writing and editing the manuscript. Figures have been done by NR and UK. All authors contributed to the article and approved the submitted version.

## Funding

This work was supported by the ANR-13-ISV3-0009 and the French Government’s Investissements d’Avenir program Laboratoire d’Excellence Integrative Biology of Emerging Infectious Diseases (grant no. ANR-10-LABX-62-IBEID).

## Conflict of Interest

The authors declare that the research was conducted in the absence of any commercial or financial relationships that could be construed as a potential conflict of interest.
